# Oral health-related quality of life in children with osteogenesis imperfecta

**DOI:** 10.1007/s40368-021-00664-9

**Published:** 2021-11-20

**Authors:** J. M. Cachia Mintoff, M. Riddington, S. Parekh

**Affiliations:** 1 Eastman Dental Hospital, London, UK; 2 Present Address: Great Ormond Street Hospital, London, UK; 3 UCL Eastman Dental Institute, London, UK; 4 Happy Kids Dental Clinic, London, UK; 5 Present Address: CAMHS Specialist Eating Disorders Service, Kingsley Green, 15 Forest Lane, Harper Lane, Herts, WD7 9HQ UK; 6 Department of Paediatric Dentistry, Bloomsbury Campus, Rockefeller Building, 21 University Street, London, WC1E 6DE UK

**Keywords:** Community paediatric dentistry, Disturbances in dental development, Medically compromised/disability

## Abstract

**Purpose:**

Osteogenesis imperfecta (OI) results from mutations in the genes involved in the modification or biosynthesis of collagen. This study aimed to assess the oral health-related quality of life (OHRQoL) in children with OI.

**Methodology:**

Participants were recruited from a highly specialised OI centre for children. The Child Oral-Health Impact Profile—Short Form (COHIP-SF) was used, adding demographic and qualitative questions. Children aged 8–16 years participated between January and October 2019. Statistical analysis was carried out. A higher COHIP-SF score indicates better OHRQoL (maximum score, 76).

**Results:**

One hundred and six (106) children participated (44 female, mean age 11.93 years). COHIP-SF median score was 59. Children reporting mild OI (*n* = 55) had higher median scores (62) compared to severe OI (*n* = 7) with median scores of 55 (*P* = 0.087). When comparing mixed (< 12 years, *n* = 46) and permanent dentition (≥ 12, *n* = 60), no significant difference in OHRQoL was seen (*P* = 0.977). There was no significant difference between severities for each COHIP-SF domain. Limited data on the presence of dentinogenesis imperfecta did not impact overall score (*P* = 0.109), but was significant in the oral-health domain (*P* = 0.033).

**Qualitative:**

Common themes were the need for braces, discolouration, pain and function.

**Conclusion:**

This study confirmed that children with OI have dental concerns in areas including oral health, functional well-being and socio-emotional well-being. This was related to severity of OI.

## Introduction

Osteogenesis imperfecta (OI), also known as ‘brittle bone disease’ (Forlino et al. 2011), is a genetic condition resulting from a mutation in the genes involved in the modification or formation of collagen (Byers et al. 1989). It affects type I collagen, and can be associated with dentinogenesis imperfecta (DI), a genetic disorder of teeth (Okawa et al. 2017).

The prevalence of OI varies between 0.3 and 1.5 in 10,000 live births (Stevenson et al. 2012; Folkestad et al. 2016; Kuurila et al. 2002). Different subtypes exhibit varying degrees of bone fragility (Okawa et al. 2017). The first classification for OI was introduced in 1979 and included four subtypes of OI of autosomal inheritance and attributed to mutations in COLA1 and COLA2 (Sillence et al. 1979). Later, other subtypes were added due to the genetic, clinical and radiographic variability seen in the types of OI (Van Dijk and Sillence 2015). Genetic classification now includes 20 subtypes (Marini et al. 2017). Diagnosis of OI is made based on several parameters. These include clinical evaluation of a child who has several bone fractures early in life, family history of OI, radiographic appearance and genetic testing (Marini et al. 2017). The clinical features of OI are related to collagen abnormalities and include hearing loss, which can be seen across all types of OI and tend to be a progressive disorder due to sensorineural and conductive deficiencies (Forlino et al. 2011): blue sclera, which results from the abnormal way the light reflects off the collagen in the sclera (Pillion et al. 2011); and neurological features including macrocephaly, syringomyelia, basilar invagination and hydrocephalus (Forlino et al. 2011; Brizola et al. 2017). The most significant of the neurological features is basilar invagination which can lead to brainstem distortion due to an infolding of the skull base (Forlino et al. 2011). Another typical feature is short stature, which may be due to:

o scoliosis, vertebral and long-bone fractures, kyphosis and bone deformities;
o matrix and cellular abnormalities associated with OI; or
o a reduced responsiveness to growth hormone (Jain et al. 2019).

Wormian bones of the skull are another feature and are visible on radiographs (Dahan-Oliel et al. 2016). Other features include hypermobility, which can lead to unstable knees and feet due to knee hyperextension, flat feet and hip extra-rotation (Monti et al. 2010); respiratory and cardiac abnormalities, fatigue (Forlino et al. 2011) and obesity (Chagas et al. 2012). Dental concerns are also features of OI, most commonly including dentinogenesis imperfecta (Rousseau et al. 2018), malocclusions (Okawa et al. 2017; Rizkallah et al. 2013), posterior crowding and ectopic or impacted teeth, and a possible increase in the incidence of caries (Schwartz et al. 1984).

Oral health-related quality of life (OHRQoL) has been defined as ‘a multidimensional construct that reflects (amongst other things) people’s comfort when eating, sleeping and engaging in social interaction; their self‑esteem; and their satisfaction with respect to their oral health (Bennadi et al. 2013). Oral care impacts quality of life, as people frequently visit the dentist to alleviate pain or improve aesthetics (Bagramian et al. 2002). Differences have been noted between oral health-related quality of life in children and in adults, therefore it is important to assess OHQoL in children (Genderson et al. 2013).

The importance of evaluating OHRQoL for OI is gaining recognition, as evidenced by a Canadian study from the Shriner’s Children’s Hospital (Najirad et al. 2018). This was the first study to investigate OHQoL in OI children, and the paper was published during our research period. OI is an important rare collagen disorder and due to the oral manifestations of this condition and those associated with its treatment, it is important for dentists to understand the needs of their local population to ensure appropriate dental management is provided. The aim of this study was to investigate the OHRQoL of children with OI, to better tailor our management towards their specific needs. Additionally, there was a secondary aim to assess if there was any impact on OHRQoL depending on demographic data such as age or gender and depending on the severity of participant’s OI or type of OI.

## Methodology

### Study design

This was a mixed qualitative and quantitative study using a questionnaire, for children with OI, aged between 8 and 16 years, receiving care at a highly specialised OI clinic. Participants were recruited during routine scheduled appointments at the OI clinic from 11th January to 25th October 2019. Ethical approval was obtained (reference number: 18/NS/0129) and recruitment was scheduled to run for approximately 1 year aiming to collect at least 100 valid questionnaires. Parent and child information leaflets and consent forms were developed.

#### COHIP-SF

The COHIP-SF questionnaire (Broder et al. 2012) was selected as it has been validated for children from 8 to 16 years old and used in similar studies related to conditions such as cystic fibrosis (Patrick et al. 2016). Due to its wide age range, COHIP-SF allows for comparison between age groups, unlike other questionnaires such as the CPQ which has two different questionnaires for children older or younger than 11 years. The short form of the questionnaire was chosen to ensure the children participating were not overwhelmed by the number of questions and could therefore answer the questionnaire quickly and without too many problems (Broder et al. 2012).

The COHIP-SF consists of 19 questions divided into three domains: oral health, functional well-being and socio-emotional. Each domain reflects an aspect of the child’s life which will contribute to their overall OHRQoL. The maximum achievable OHRQoL score is 76, indicating a higher OHRQoL. The questionnaire asked the participant to consider each question in light of how they felt in the 3 months preceding the questionnaire.

#### Qualitative questions

Two qualitative questions were added at the end of the questionnaire to gain further insight into how the participants felt about their teeth and what changes they would make if any. These questions were analysed using thematic analysis and a framework type approach and responses were grouped into themes.

#### Young Persons Advisory Group

To ensure adequate understanding and readability of the documents to young participants, these were shown to the Young Persons Advisory Group (YPA group) at the hospital in July 2018. The YPA Group is a group of young persons aged between 9 and 20 years who hold meetings once a month and give feedback to researchers carrying out medical research involving children.

They gave useful feedback on the information leaflet, generally agreeing that whilst easy to read and understand, a second information leaflet should be developed for slightly older children with more mature language. Their feedback resulted in the development of a second children’s information leaflet for children aged 13–16 years. The original information leaflet was targeted towards children aged 8–12 years.

### Setting

Participants were approached during their routine OI checkups (occurring annually or biannually depending on the severity of their OI) at a highly specialised OI service in London. Data collection started on the 11th January 2019 after obtaining ethical approval. All participants and their families were supplied with information leaflets and the study, what it entailed and its aims were explained by the primary investigator prior to the participants agreeing to take part in the study. Participants and their families were given as much time as needed to make the decision and any relevant questions were answered by the primary investigator. Participants were approached until 25th October 2019.

### Participants

#### Inclusion criteria

- Children with OI.
- Children between the ages of 8 and 16 years.
- Children capable of understanding the questionnaire.
- Parental consent given.
- English speaking participants or those for whom a translator was present at appointment.

#### Sample size

According to the records at the highly specialised OI centre, 172 of their OI patients fit the inclusion criteria. For a confidence interval of 90%, a margin of error of 5% and a response distribution of 50%, the required sample size was 106 responses in a population of 172. The plan was therefore to recruit approximately 100 participants to the study.

### Data collection

Participants fulfilling the inclusion criteria and their legal guardians were given information sheets by the primary investigator and OI team and written informed consent was obtained. Paper copies of the questionnaire were handed out to the participants and the completed forms were placed in a sealed envelope. Once the completed consent forms and questionnaires were returned, the cover page of the questionnaire and the consent forms were separated from the rest of the questionnaire to maintain anonymity. The questionnaires and consent forms were stored separately at secured locations.

### Statistical analysis

All data was inputted into MS Excel and statistical analysis was carried out using both MS Excel and SPSS. Analysis included simple descriptive statistics, tests to assess for type of distribution and a combination of parametric (*t* tests) and non-parametric tests (Mann–Whitney *U*) were used for the questionnaire data. Non-parametric tests were used to analyse those data sets which were not normally distributed. Thematic analysis (framework analysis) was used for the qualitative questions to obtain meaningful results.

## Results

A total of 106 questionnaires were collected during the study period. Three families refused to answer the questionnaire for the following reasons:

- In one family the daughter refused to answer.
- In the second family the parents were unwilling to spend the time to participate.
- The third family did not give a reason.

Table 1 shows the demographic data for the participants including age, gender, ethnicity, self-reported severity of OI, type of OI and DI status. Respondents’ self-reported ethnicity was grouped according to the Office of National Statistics Groupings (Office of National Statistics, 2016). The majority of the participants self-reported as white —54% (*n* = 57). Two-thirds of the children participating (66%, *n* = 70) reported their OI type. For children who did not know what type of OI they had, the information was obtained from their medical records. With respect to the subjective severity of OI, the majority of cases self-reported as mild (52%, *N* = 55), which corresponded well with type 1 OI. Only 7% (*n* = 7) of participants self-reported their OI as severe.

**Table 1:** Demographic information and clinical conditions of the sample

	Overall	
	Numbers	Percentage
Numbers	106	100%
Age		
Average age	11.93	n/a
Age range	8–16	
Less than 12 years old	46	43%
12 years old or older	60	57%
Ethnicity		
Asian	14	13%
Black	7	7%
Mixed	3	3%
White	57	54%
N/A	16	15%
Other	9	8%
Self-reported severity of OI		
Mild	55	52%
Mild to moderate	27	25%
Moderate to severe	11	10%
Severe	7	7%
N/A	6	6%
Type of OI		
Type I	52	49%
Type III	8	8%
Type IV	21	20%
Type V	3	3%
Other types [VIII, XI, XVII]	3	3%
Unconfirmed if having OI	4	4%
Unknown type	15	14%
DI status		
Has DI	14	13%
Does not have DI	34	32%
Unknown	58	55%

### COHIP-SF questions

Internal consistency was checked by calculating Cronbach’s alpha [0.821]. Less than 1% of questions were not answered. Missing answers did not appear to show a bias towards age, gender or ethnicity; however, the number of missing answers in this section was too small for significant results.

Normality tests (Shapiro–Wilk/QQ plots) found normal distribution for the oral health well-being domain and non-normal distribution for:

- Overall COHIP-SF.
- Functional well-being domain.
- Socio-emotional well-being domain.

#### Overall COHIP-SF

Comparisons for gender, age groups (younger and older than 12 years), ethnicity, self-reported severity, type of OI and absence or presence of DI, showed that none were statistically significant. When comparing those with self-reported mild OI to those with self-reported severe OI there was no significant difference (*P* value 0.087). Males scored slightly better overall and those with mild OI had higher scores than those with severe OI (Table 2). As the data was not normally distributed, the median and interquartile range and calculated for data analysis.

**Table 2:** Showing overall COHIP-SF values and *p* values (data not normally distributed)

	Numbers	Median	Interquartile range	*P* value
Overall COHIP-SF	106	59	15	
	Range 19–73			
Gender				
Male	62	56.5	15.3	0.16
Female	44	60.0	12.5	
Age				
Younger than 12	46	58.0	13.0	0.98
12 years old or older	60	59.0	16.5	
DI status				
Has DI	14	60.5	17.5	0.11
Does not have DI	34	56.5	9.0	
Unknown	58	57.5	15.3	
Ethnicity				
White	57	58	14.25	Mixed ethnic group vs Asian group 0.05 (Significant)
Multiple/mixed ethnic group	3	69	4	
Asian/British-Asian	14	49.5	21.25	
Black/Caribbean/black-British	7	57	8.5	
Other	9	62	4	
No answer	16	58	13.25	
Subjective severity of OI				
Mild	55	62.0	12.0	Mild vs severe OI – 0.087
Mild to moderate	27	59.0	14.5	
Moderate to severe	11	57.0	11.5	
Severe	7	55.0	18.0	
No answer	6	49.5	2.5	
Type OI				
Type I	52	59.0	15.5	Type V (highest scoring) vs unclassified type (lowest scoring) − 0.614
Type III	8	58.0	10.8	
Type IV	21	57.0	14.0	
Type V	3	67.0	6.5	
Other [types VIII, XI, XVII]	3	55.0	12.0	
Unconfirmed	4	60.0	11.0	
Unclassified type of OI	15	48.5	8.0	

The question, *‘Have you ever felt that you were attractive *(*good looking) because of your teeth, mouth, or face?’* scored consistently low with 35% (*n* = 37) choosing the lowest scoring response. There was no bias towards age for these respondents; however, 70% of those who chose the lowest scoring response were males. Another question which achieved low overall scores was *‘Have you ever had crooked teeth or spaces between your teeth?’ with 29% *(*n* = *31) choosing ‘almost all of the time’.* There appeared to be no bias towards age or gender for those who responded with the lowest scoring response.

Questions which scored consistently high with over 60% of participants claiming they had never felt this way included: [Have you ever…].

- *been unhappy or sad because of your teeth, mouth or face?*
- *avoided smiling or laughing with other children because of your teeth, mouth or face?*
- *had trouble sleeping because of your teeth, mouth or face?*
- *been teased, bullied or called names by other children because of your teeth, mouth or face?*

There did not appear to be any bias towards age or gender with the above questions.

#### COHIP-SF domains

A summary of the key results in each domain can be found in Tables 3, 4, 5.

**Table 3:** Key results and statistical significance in the oral health well-being domain

Distribution	Normal
Mean (SD)	12.60 (3.59)

	*P* value	Significant or not significant
Gender	0.161	Not significant
Age groups	0.083	Not significant
Severity	0.125	Not significant
type of OI	0.206	Not significant
Ethnicity	0.085	Not significant
DI	0.033	Significant

**Table 4:** Key results and statistical significance in the functional well-being domain

Distribution	Not normal
Median (IQR)	14 (4)

	*P*-value	Significant or not significant
Gender	0.719	Not significant
Age groups	0.407	Not significant
Severity	0.335	Not significant
type of OI	0.043	Significant
DI (overall)	0.982	Not significant
DI (under 12 years old)	0.039	Significant

**Table 5:** Key results and statistical significance in the socio-emotional well-being domain

Distribution	Not normal
Median (IQR)	31 (7.75)

	*P* value	Significant or not significant
Gender	0.400	Not significant
Age groups	0.073	Not significant
Severity	0.105	Not significant
type of OI	0.114	Not significant
DI	0.069	Not significant

#### Oral health well-being domain

In the oral health well-being domain (normally distributed), the mean score was 12.60 out of 20 (SD 3.59, range 4–20). There was no significance between gender (*P* = 0.161), age groups (*P* = 0.083), severity (*P* = 0.125), type of OI [type I vs type V *P* = 0.206] or ethnicity (*P* = 0.085). There was a significant difference in the oral-health well-being domain between those with and those without DI (*P* value 0.033).

#### Functional well-being domain

For functional well-being (non-normally distributed), the median score was 14 (interquartile range = 4, range 3–16), and there was no significant difference between genders (*P* = 0.719), age groups (*P* = 0.407) or severity [mild vs severe *P* = 0.335]. There was also no significant difference between those who had DI and those who did not; however, those having DI scored considerably lower than those without (*P* = 0.982). For those who were under 12, there was a significant difference between those who had DI and those who did not (*P* = 0.039). For those 12 or older, there was no significant difference (*P* = 0.164). Type V OI had the highest median score (16) in this domain, whilst the lowest scores were for the other types of OI [types VIII, XI and XVII] with a median of 10. The difference between these two groups (types V and other types) of OI was statistically significant (*P* = 0.043). The numbers in these groups (types V and other types) were small and need to be interpreted cautiously.

#### Socio-emotional well-being domain

For the socio-emotional well-being domain (non-normally distributed), the median score was 31, (interquartile range = 7.75, range 4–40). Again, there was no significant difference for gender (*P* = 0.400), age groups (*P* = 0.073), DI status (*P* = 0.381) and severity of OI (*P* = 0.105). There was a greater statistical difference between the severe and moderate to severe scores; however, this again was not statistically significant (*P* = 0.069).

Once again, the type V OI group had the highest median (37) score from the types of OI in this domain. The lowest scores were in the group who were unconfirmed as having OI with a median of 28. The difference between these two groups of OI was not statistically significant with a *P* value of 0.114. Again, the numbers in these groups (types V and unconfirmed to have OI) were small and need to be interpreted cautiously.

#### Qualitative questions%

Two optional qualitative questions ended the questionnaire:

1. *‘What one change to your teeth or smile would make the biggest difference in your life? And how would things be different for you?’.*

2. *‘Is there anything else about your teeth, mouth or face that you think is important? Please tell us what it is.’*

The first question was answered by 61% of participants (*n* = 65) and thematic analysis revealed five major themes (Table 6). When comparing COHIP-SF scores of those participants who responded to the first qualitative question and those who did not, those responding had a lower median COHIP-SF score (55) than those who did not (60) and the difference was significant (*P* = 0.035).

**Table 6:** Showing themes picked out from question ‘What one change to your teeth or smile would make the biggest difference in your life? And how would things be different for you?’

Theme	Quote
Aesthetics Subdivided into: Orthodontics, discolouration, both orthodontics and discolouration, others	*‘To make them less crooked, gappy and align all my teeth’*
Confidence	*‘To make my teeth connect properly. It would be different because I would be more confident in smiling’*
Function	*‘Healthy and strong teeth would be very important in order to be able to eat what I like and not have to worry about damaging my teeth’*
Hygiene	*‘To be cleaner’*
Pain or caries	*‘I don’t want fillings so teeth don’t hurt, I want my teeth to grow quicker. I want my side tooth to grow straight so I don’t have weird teeth’*

The second question was less well responded with 78% (*n* = 83) not answering the question. Thematic analysis identified five themes (Table 7). Again, there was a significant difference between COHIP-SF scores of those who responded and those who did not (*P* = 0.030) with those responding having a lower COHIP-SF score.

**Table 7:** Showing themes picked out from question ‘Is there anything else about your teeth, mouth or face that you think is important? Please tell us what it is.’

Theme	Quote
Aesthetics	*‘Brings my chin forward and looks displeasing when I smile’*
Hygiene	*‘Cleaning properly’*
Pain or caries	*‘Pain, very much pain’*
Confidence	*‘That you see feel good about them’*
Other	*‘Gag reflex’*

## Discussion

The importance of evaluating OHRQoL for children with OI is gaining recognition around the world (Najirad et al. 2018). To the best of the authors’ knowledge, this is the second article to be published on this topic. In this discussion we compare our results to those of Najirad et al. (2018).

Males and females are equally likely to be affected by OI (Dahan-Oliel et al. 2016). This was reflected in our sample of OI patients, 58% of whom were male. The rarer types of OI (types V–XX) make up around 21% of cases (Marini et al. 2017), whereas in our sample it made up only 6%. However, these discrepancies may be because of the small sample size (*n* = 106) and those with unclassified or unconfirmed OI making up 18% of our cohort.

OI has not been seen to discriminate between ethnicity and race (Marini and Smith 2000). In this study, 69% of participants were identified as white. When comparing this proportion to the United Kingdom (UK) it showed a high proportion of ethnic minority groups in this study. In the UK the proportion of people who do not identify as white is 13%, compared to 31% in this study (Office of National Statistics, 2016). The large proportion of minority groups may affect the overall results of the questionnaires, as people from different ethnicities may have different ideas or priorities on what affects their quality of life.

In this study, 13% of the participants had dentinogenesis imperfecta (DI) which is lower than the overall prevalence of DI in children with OI (22–25%) reported in recent studies (Najirad et al. 2018; Hald et al. 2018). However, there was missing data on DI, so this proportion must be interpreted cautiously. The proportion of participants with type III OI having DI was 38% and the proportion of those with type IV OI having DI was 29%; however, there was missing data on the presence of DI in both groups and the number of participants with type III OI (*n* = 8) and type IV OI (*n* = 21) was small and needs to be interpreted cautiously. It appeared that DI did not significantly impact overall OHRQoL scores. However, when analysed according to domain, those with DI felt they had a worse OHRQoL in the oral-health well-being domain. This could be due to awareness of aesthetic differences, pain or sensitivity or increased treatment need. A significant difference was also seen in the functional well-being domain between those under 12 years with DI and those under 12 years without DI. Again, this would make sense as DI is more common in the primary dentition and the loss of tooth structure may have an effect on the oral functioning of these children. This needs to be viewed with caution because there were only 14 participants who had DI.

Two questions of particular importance for dental issues in this population are ‘Have you ever had crooked teeth or spaces between your teeth?’ and ‘Have you ever had discoloured teeth or spots on your teeth?’, as children with OI commonly have DI and malocclusion. For both questions, the majority of children felt they had discoloured (50%) or crooked or spaced teeth (65%) at least some of the time and this is relevant as it shows that children with OI are aware of their dental health and it can affect their OHRQoL. Assessing children with OI for malocclusion and discolouration and treating it when it causes concerns can help to increase the OHRQoL in this cohort.

It reflects well on our society that 86% of the participants felt they have ‘never’ been bullied, and none of the children felt they were bullied ‘often’ or ‘almost all of the time’ because of their face and mouth.

A 9-year-old boy with self-reported mild OI (type I) had the worst OHRQoL with a score of 19. This was unexpected and shows that quality of life is subjective and is not necessarily related to severity or type of OI. He also reported having discoloured teeth ‘almost all of the time’. This suggests that whilst he did not have DI, he may have had another dental anomaly causing discolouration which was affecting his OHRQoL such as enamel defects. Ideally, interviews or focus groups would have given a more definitive answer.

It was interesting to see that self-reporting did seem to correlate with severity. For example, 75% of children with type I OI said they had mild OI, and likewise the majority of children with type III reported severe OI. To our knowledge, there are no other studies correlating patient-perceived severity of OI to type of OI. This is a gap in the literature which may impact both OHRQoL and general QoL. Generally, those with mild OI had a better, but insignificant, overall COHIP-SF score than those with severe OI. It was surprising that there was no significant difference between those with mild and severe OI and this shows that severity of OI may not necessarily relate to severity of dental concerns. Children with OI have other complex social factors affecting their life such as their ability to play with their peers, frequent visits to hospitals or clinics. Fear of fractures or feeling a lack of independence may colour the way a child perceives their OHRQoL, particularly if they feel different from their peers and this may account for why severity of OI is not significantly correlated to overall OHRQoL.

The two qualitative questions were asked to further explore the participant’s main concerns and what, if anything, they would change about their oral status. It was noted that that those with lower COHIP-SF scores, and therefore a lower OHRQoL, were more likely to comment on the reasons for their lower OHRQoL than those participants who had a higher COHIP-SF score and therefore a better OHRQoL.

A recent Canadian study (Najirad et al. 2018) assessed 138 children with OI recruited from the Brittle Bone Consortium over 2 years using the Child Perception Questionnaire (CPQ) for ages 8–11 and 12–14. Severity was assumed depending on the type of OI. The Canadian study found a higher prevalence of DI in their studied population (22%) compared to this study (13%). Najirad et al. also found that older children with severe OI had poorer OHRQoL than older children with mild OI (Najirad et al. 2018). Our study did not find significant results between the mild and severe types of OI in the older cohort. We were unable to compare age groups in this study, as the CPQ questionnaires are different for ages 8–11 and 12–14. Gender was not mentioned as a factor influencing OI in the Canadian study (Najirad et al. 2018).

Although data are still limited, information on health-related quality of life (HRQoL) in children with OI is increasing. Studies have found that children with more severe types of OI (types III and IV) had significantly lower HRQoL scores than those with milder (type I) OI. They also found that children with OI scored lowest in the physical domains of HRQoL tools (Dahan-Oliel et al. 2016; Song et al. 2019). Comparing these to our study, we see that whilst children with types III and IV OI (severe) did score lower than those with type I OI (mild), the difference was not significant. This is likely due to the small number of participants with either type III or IV OI (*n* = 29) compared to those with type I (*n* = 52).

A limitation of this study is the lack of data available regarding the presence of DI for 55% of the participants. Therefore, the analysis should be interpreted cautiously. Ideally, we should have asked participants whether they had DI or not. Alternatively, clinical examination could have indicated the presence of DI or other dental anomalies.

Another limitation was the small sample size of the more severe types of OI such as type 5; here again, results will need to be interpreted cautiously. A larger-scale project on a national or international level can give more significant information on the less common type of OI.

Furthermore, this study did not ask patients whether they were undergoing bisphosphonate therapy or not. This treatment can impact their oral health and how they perceive it and may have an impact on the results obtained.

The present study aims to benefit the OI community as it disputes the assumption that clinical severity of OI is linked to OHRQoL and therefore overall quality of life. It shows that dental care should be tailored to each child on an individual basis depending on their oral condition. The results of the COHIP-SF reinforce that it is not only a child’s general health, but also their oral health which impacts their quality of life. Additionally, the need to educate general dentists and orthodontist on the importance of treating children with OI or referring to an appropriate specialist early on is clear.

## Conclusions

Within the limitations of the present study, the following findings were revealed:

- Oral health-related quality of life (OHRQoL) is highly subjective and whilst children with self-reported severe OI had worse OHRQoL scores than those with mild OI, the difference was not significant.
- Age and gender were not indicators of better or worse OHRQoL in children with OI.
- The presence of DI was significant in the oral health well-being domain, and in the functional well-being domain for those children younger than 12 years.
- A low score in the socio-emotional domain was an indicator of worse overall OHRQoL.
